# The Recent Review of Swine-Disease Literature

**Published:** 1890-01

**Authors:** D. E. Salmon


### THE RECENT REVIEW OF SWINE-DISEASE LITERATURE.

*To the Editor of* THE JOURNAL OF COMPARATIVE MEDICINE AND VETERINARY ARCHIVES:

*Sir:* The attack which was made on me in the pages of the October number of the Journal under cover of an alleged review of the recent literature bearing upon epizootic swine-diseases, cannot be allowed to pass entirely without notice. It is the custom of authors, and one which I have generally followed, to allow the reviewer unquestioned liberty in criticising the literary or scientific merits of a publication. But when the reviewer goes beyond this and begins his criticism by a positive announcement of the "official incompetency and insincerity" of an author, without producing any tangible evidence to sustain his accusation, he is taking a position where he can no longer expect the usual courtesies observed among authors. With the sincerity of the reviewer I shall have nothing to do. He may believe every word he writes, and he may be another Guiteau who thinks himself divinely appointed to accomplish some extraordinary mission. My duty ends with showing that he is ignorant of his subject, that his alleged facts on which his argument is based have not been verified by him and are grossly incorrect statements, and that the scientific questions have been examined by competent scientists in Europe aud America, whose conclusions are diametrically opposed to those which he promulgates with such positiveness. In his personalities he has not confined himself to a single individual. The three eminent scientists appointed on a Board of Inquiry to decide the points in dispute are dismissed as writers of a "whitewashing report," and, according to him, "it is evident that considerable dust has been thrown in the eyes of European writers by the report of the Department (Bureau? D. E. S.) of Animal Industry." He even goes out of his way to state that the Department of Agriculture was for at least one Presidential term "under the management of a commissioner, whose honesty we were inclined to question."

Leaving these personalities, which neither add to the value of the review nor to the reputation of the reviewer, we will now examine in detail some of the assertions from which the deductions are made. The reviewer first goes back to Special Report No. 22, published in 1880, and quotes a paragraph to show its unscientific character, but he refrains from adding, that the letter from which he quotes, was not written by an employé of the Department, but by a physician in Mississippi, and was probably printed to show the current medical opinion in regard to the prevailing diseases of animals in that section.

He immediately repeats this blunder by quoting from the same volume what he is pleased to designate an "auto-review" of "Special Report No. 12." "An indignant and amused colleague," he says, "called our attention to the fact that it was chiefly directed against Dr. Detmers, who had the good or bad fortune not to be a government pensioner at the time. That veterinarian had published certain good results obtained in hog-cholera through the rational and systematic use of carbolic acid. The department pensioner and expert, however, scouts this," etc. In this case, as in the other, the letter critised was written by a physician not in the employ of the Department, and who used the most complimentary language in regard to Dr. Detmers' report, saying that it was "so very thorough in all respects" and "so generally supported by the other commissioners" that he confined his criticism to it on that account. The reviewer's statement as to who was the "department pensioner" is exactly the reverse of the truth. Dr. Detmers was at that time, and had been since 1878 a regular employé of the Department. Dr. McGehee, who wrote the letter in question, was in no way connected with the Department.

"A little later," we are told, "the Department of Agriculture lent itself to a very questionable aim, in furthering, or at least tacitly endorsing, a crusade against Dr. Detmers, when he was accused of having falsely started a pleuro-pneumonia scare." It is difficult to understand what is meant by "furthering or tacitly endorsing a crusade against Dr. Detmers." The fact is that Dr. Detmers was charged with two inexcusable blunders in stating that pleuro-pneumonia existed in the West—one of these was said to have occurred in the Chicago Stock Yards and one in Iowa in 1887. In both cases there had been an opportunity for *post-mortem* examinations. But the Department, so far from yielding to the demand for his dismissal, retained him in its employ until 1884. If this can be called furthering or endorsing a crusade against a man it is a curious way of accomplishing such an object.

Omitting a few paragraphs for the present we reach what purports to be a brief "history of hog-cholera in the United States." We are told that in 1878 "the Government appointed two veterinarians, Detmers and Salmon, to investigate the subject." This statement, while literally correct, is misleading. Three veterinarians and six physicians were appointed for two months in 1878. The time of Drs. Law and Detmers was extended, and their investigations were continuous, while a whole year passed before Dr. Salmon was re-employed. This fact, lost sight of by the reviewer, makes an important difference in estimating the work done at the time these early reports were written.

The reviewer then goes on to say that, "In the report of 1880–81, Dr. Salmon announces a micrococcus as the cause of swine-plague." There is no such announcement made in that report. Turning to p. 69, my exact language is found as follows: "I had hoped by cultivation experiments to prove that the granules observed, either were the cause of the disease or that they were an epiphenomenon; but owing to the fact that the virus in every case lost its activity after the first generation, or became too mild to afford satisfactory results, such evidence could not be obtained." In the report for 1881–82, and in that for 1884, I published experiments which led me to believe that the cause of swine-plague was a micrococcus, and I only make the above quotation to show the inaccuracy of the reviewer.

We now come to what is evidently considered the *piéce de résistance* of the whole review. It reads: "In connection with these facts the following admission made by Salmon has a very strange appearance. In speaking of the association of Dr. Smith in his work, he says, that he made some slight changes in the latter's report: a phrase reading 'we no longer consider a micrococcus as the cause of swine-plague' to 'we no longer consider a micrococcus as the cause of all outbreaks of the disease known as swine-plague.' Now it is only too evident that by a sinister coincidence, while Dr. Salmon was preparing to abandon the micrococcus and return to the bacillus (without any disfiguring reference to the real discoverer) he claimed the existence of two diseases among swine, calling one 'hog-eholera' and the other 'swine-plague.' Through all of these gyrations and evasions Dr. Billings hunted Salmon with the keenness of a medical Nimrod.

Now, to get at the facts, it must be remembered that the admission which the reviewer considers so strange was made in regard to the report of 1885, and this was written before Dr. Billings began his investigation of swine diseases, and at a time when he was an applicant for appointment in the Bureau of Animal Industry, and was writing complimentary remarks about these very investigations. All of his Nimrod business was subsequent to this, beginning after he failed to secure the coveted appointment, and, consequently, has no bearing upon the quotation we are considering. With this irrelevant material out of the way, I should like to ask the scientific reader what there is strange in my making the correction above referred to? Consider the circumstances. I had been investigating swine-diseases and had found a micrococcus which gave me positive results when inoculated in swine. Dr. Smith was afterwards associated with me, at my request, and was placed by me in charge of the bacteriological work of the Bureau. Soon after this he found in an outbreak of swine-disease a very different germ, a bacillus, which we proved to be the cause of the outbreak. It being his first investigation of swine-diseases, he was probably inclined to look upon this discovery as solving the whole question. There evidently was another conclusion which was equally likely to be correct, viz., that we had been confounding two distinct diseases together, as had been done in Europe with charbon and black quarter. As my own investigations supported this conclusion, was it not my duty to maintain this possibility until further researches definitely settled the whole question? At any rate the conclusion of that report carried with it the influence of my opinion as chief of the bureau, and it was only proper that it should represent my views.

I may add here, that while the investigations have been directed by me, and have been carried out according to my plans, all the reports from 1885 to 1889, inclusive, so far as they relate to experiments, bacteriological observations and *post-mortem* notes have been written by Dr. Smith; and consequently all the criticisms of their contents aimed at me, personally, fall flat,.

because I am neither their author nor have I made any changes in the record of observations. I firmly believe, however, that they are as accurate as it is possible to make such reports, and I know that they have done more to clear up the difficult questions relating to swine-diseases in this country than all other work combined.

But to return to our reviewer: "Now, it is only too evident that by a sinister coincidence," he says, "while Dr. Salmon was preparing to abandon the micrococcus and return to the bacillus (without any difiguring reference to the real discoverer), he claimed the existence of two diseases among swine, calling one 'hog-cholera' and the other 'swine-plague.'" As a matter of fact the distinction between hog-cholera and swineplague was not made in the report for 1885, and not until the following year. So that the sinister coincidence only occurs in the reviewer's imagination. In 1886, Dr. Smith re-discovered the other disease, and we were able to satisfy ourselves by experimental evidence that there were two separate and distinct diseases. The only question that can be raised is as to whether the microbe of swine-plague is identical with the micrococcus described by me in previous reports. Germs of this variety are now called by most observers bacilli, but previous to 1885 they were generally called micrococci. Morphologically the microbe is identical with the fowl cholera germ which I at the same period called a micrococcus, as did also nearly all bacteriologists of the time. This explains the only apparent discrepancy, and it makes little difference to me whether the explanation is accepted or not, as without it we still have a priority of discovery over any American investigators.

Now a word as to the discovery of the hog-cholera bacillus by Dr. Detmers. That this gentleman described bacilli in his reports is true, but it is equally certain that he saw and figured several very different species, that none of them were described with sufficient accuracy to enable any one to identify them, and that none of them were connected by experimental evidence with the causation of the disease. It is also certain from his own report that when he received a culture of the true hog-cholera bacillus he failed to recognize it, and claimed that it did not produce the same disease which he had studied as hog-cholera, (Fifth Annual Report, Ohio Agricultural Experiment Station, 1886, pp. 289–290.) Klein had attributed the swine-fever of England to a bacillus several months previous to Detmer's first investigations for the Department, and like the latter gave a description which does not apply to the true germ. Just how much credit if any should be given to these gentlemen for this work I am willing to leave unbiased scientists to decide. To my mind it is not the discovery of germs in the liquids of affected animals which entitles a man to credit, but it is the isolation of the pathogenic species, its accurate description and the production of experimental evidence to show that it causes the disease. And no work of this kind was done by Detmers, if we except an attempt to make cultures in unsterilized milk, using ordinary bottles instead of culture tubes, and starting his cultures with material obtained from a putrid ulcer on the internal surface of the stomach. It is probable that not even the reviewer would care to hold up the details of such work to the scientific world and demand credit for it.

The two chief conclusions of the reviewer in regard to the work of the Bureau are very curious. He says: "On carefully reviewing the present state of our knowledge on the subject of American swine-plague, it appears evident to us, 1st, that Salmon's micrococcus is not and never was a specific disease germ of anything. Whether created in the manner suggested by Billings, or due to the adoption of erroneous methods, it will disappear, if it has not already disappeared from the columns of trustworthy bacteriological literature."

On the other hand his protegé, Billings asserts "That the object described by Salmon as the germ of hog-cholera cannot be cultivated from the tissues of any animal that has died of hog-cholera or swine-plague," (Report on Swine-Plague, p. 74.) Between the two, therefore, it would appear that the germs described by the Bureau of Animal Industry, as found in epizootic swine-diseases in this country, can meet no better fate than ignominious extermination at the hands of these writers. If they had both hit upon the same germ "as created in the manner suggested by Billings," that is, by the process of "forgery," whatever that word may mean in bacteriology, we might hope to obtain eventually some sort of a consistent statement as to their views; but as it is, the more they explain the harder it is to understand what they mean.

The second conclusion is worthy to stand by the first. It reads, "2d. That while Salmon in 1886 claimed swine-plague to be a distinct specifie disease, characterized by pneumonia, his hog-cholera was described as an equally specific disease characterized by an equally characteristic enteritis, he totally abandoned this position in 1887." It would puzzle even the reviewer to quote any sentence or paragraph in the report of 1887 in which there was any abandonment of such a position. On the contrary, several pages (Annual Report Department of Agriculture, 1887, pp. 513 to 519), were devoted to an analysis of the *post-mortem* notes and to pointing out the differences in the lesions of the two diseases. We did not in that report, nor have we anywhere, abandoned the position that the most prominent and most constant lesions of hog-cholera are found in the intestines, and that the most prominent and most constant lesions of swine-plague are found in the lungs. The lesions differ in different individuals, and particularly in those which come from different outbreaks, but if my reading of pathology teaches anything it is that this is equally true of other diseases. When the report for 1886 was written we had studied but a comparatively few cases of swine-plague, and had not encountered pronounced intestinal lesions. Later, we found more virulent outbreaks in which the germ had penetrated to all parts of the body, and had caused marked intestinal lesions. These differed from the lesions found in hog-cholera, and we pointed out these differences in the 1887 report. How this can be distorted into an abandonment of our position it is difficult to see. If this idea of the reviewer is correct, the only consistent course for an investigator is to write up a disease from the first *post-mortem* he makes, and never attempt to learn anything from other cases or other outbreaks, for if he adds anything to his first descriptions he is guilty of inconsistency and of abandoning his former position. Fortunately, the real scientists of the present day do not follow the reviewer's method.

The section in which the reviewer treats of the recent European literature of epizootic swine diseases is the most biased account we have yet seen, not excepting the writings of Billings himself. He takes as his authorities Baumgarten and Hess, neither of whom have made any reputation either as bacteriologists or even as investigators of swine-diseases, and he leaves entirely out of consideration such well known authorities as Nocard, Duclaux, Degive, Schütz, Semmer and Noniewicz.

Hess makes two mistakes which the reviewer, on account of his unfamiliarity with bacteriology and comparative pathology, is unable to detect. In the first place he makes a mistake in considering hog-cholera and *Schweineseuche* as identical diseases. To be sure, he quotes me as his authority that hog-cholera is "not identical with bacillary erysipelas but with swine-plague," but in this he is not correct. I did show that hog-cholera was not idenical with erysipelas, but the remainder of the conclusion was reached by the German writer without my assistance, probably because he only recognized the two diseases. In the second place he makes a mistake in identifying the bacillus of swine-fever and hog-cholera with the microbe of *Schweinesuche*. Klein's and Detmers' description of the bacillus of swine-fever and swine-plague respectively, upon which his opinion appears to be founded, cannot for a minute be taken by any one familiar with the different forms of microbes, for a description of the *Schweinesuche* germ, although they do not, if we except Klein's last article, come any nearer to the germ of hog-cholera.

The question at issue is whether there are two or three diseases which have been heretofore grouped together in Europe under the terms *rouget* and *Rothlauf* (erysipelas). The reports of the Bureau of Animal Industry first took the position that there were three, but Baumgarten, Hess, Billings and the reviewer insist that there are but two. Now what evidence have we on which to decide the question?

First we have Nocard, who tells us that he has recently investigated three outbreaks of swine-disease, and that he considers it certain that there are three distinct disease, viz: 1, Erysipelas (*rouget*); 2. Hog-cholera; 3. Infectious pneumonia or *Schweineseuche*, (*Recueil de Médecine Vétérinaire*, 1888, Jan. 15, pp. 6–9.)

Then we have Duclaux, the editor of the *Annales de l'Institut Pasteur*, who does hot hesitate to decide in favor of three distinct diseases. (*Annales de l'Institut Pasteur*, 1888, pp, 387–397.)

Thirdly, we have Ellenberger's review of the report of Schütz on *Die Schweinepest in Dänemark*, made after the latter's investigation of the *Schweineseuche* of Germany, the swine-fever of England and the *Schweinepest* of Denmark, and surely he ought to be able to give an intelligent opinion, and one which should carry great weight. Ellenberger tells us in his summary that we now know three plagues of swine: 1. *Rouget* or *Rothlauf* (erysipelas); 2. *Schweineseuche*, an inflammation of the lungs and pleuræ, with slight changes in the abdominal organs ; 3. *Schweinepest*, in which more especially the large intestine is found diseased, (*Jahresbericht über die Leistungen auf dem Gebiete der Veterinär Medicin*, 1888, p. 69).)

Fourthly, there is Degive, of Belgium, who has made independent investigations, and who gives a classification identical with that of Schütz, (*Annales de Médecine Vétérinaire*, February, 1889. Reviewed by A. Barrier, in *Rec. de Med. Vet.*, October 15, 1889.)

Fifthly, come Semmer and Noniewicz, (*Oester, Monatschr. für Thierheilkunde*, April, 1889. Reviewed by L. Moulé in *Rec. de Méd. Vét.*, October 15, 1889), who have also made Independent investigations, and who are in entire accord in their classification with Nocard, Duclaux, Schütz and Degive.

The question for the reader to decide is whether the conclusions of these investigators and eminent scientists are to be accepted, or whether the opinions of Baumgarten and Hess are to be allowed to outweigh them. I do not see how there can be a doubt, for both the quantity and quality of the evidence is decidedly in favor of three diseases.

If all of these European investigators confirm the conclusions of the Bureau of Animal Industry as to the number of diseases which exist in Europe, that must be taken as strong presumptive evidence that our investigations which revealed two diseases here, and from which the conclusion of three diseases in Europe was first drawn, are themselves correct. But we are not obliged to depend entirely upon European confirmation. We now have most excellent American authorities who hold the same views. It is fortunate that Billings clamored for a Board to decide this question, until one was appointed, for in this way he succeeded in accomplishing something which has a tendency to counteract the errors which he was led into by his unscientific methods, and which his desire for notoriety led him to immediately spread broadcast over the civilized world.

The reviewer says : "Eventually, Dr. Billings induced Congress to appropriate $30,000 to defray the expenses of an investigation into the disputed points." Dr. Billings neither induced Congress to appropriate such a sum, nor did Congress make any such appropriation. The expenses of the investigation were paid out of the lump appropriation to the Bureau of Animal Industry,

ARMY MEDICAL LIBRARY WASHINGTON, D. C. U. S. A.

and instead of there being any increased funds obtained by Dr. Billings or anybody else, the expenses for swine investigations were for the first time limited to $15,000. Here was a point which is a matter of public record, and although about fourteen months had passed since the appropriation for that year was made, the reviewer has not consulted the record, but gravely incorporates a ridiculous canard into a paper which should above all things have been correct and impartial, no matter if he felt it his duty to criticise in the most severe manner. And the more severe his criticism the more careful he should have been that his statements were correct.

In regard to this Board of Inquiry it should be stated that it was asked for by Dr. Billings to "settle the question whether there are two swine-plagues in America, or only one," Proceedings National Swine Breeders' Asssociation, 1888, p. 28), and two of the three men appointed were suggested by him as perfectly satisfactory to him for deciding this question. The third member of the board was named by me because he had been associated with Dr. Detmers in his earlier investigations, and was disposed to give him full credit for his work. As this third member has since been named by Billings in his circular on inoculation, as one of his references, and as he is the only member of the board who appears as a reference, it is to be assumed that he was not less friendly toward that gentleman than the others. In addition to this Dr. Billings published a letter in the Nebraska State Journal of December 14, 1888, in which, after giving his reason for being satisfied with each member of the board, he says: "I certainly cannot object to the commission named by Mr. Salmon."

Prof. Welch was unable to serve on the board, and was asked by the Department to name some one to serve in his place. He named Dr. Bolton, of the University of South Carolina, who had formerly been his assistant, and had worked with him in his investigation of swine-plague at Johns Hopkins University. This explains the constitution of the board, and if it was possible to show Dr. Billings any more favors in its selection, and at the same time secure impartial scientists, or if a more competent board could have been selected, I should be pleased to learn how this could have been accomplished.

In writing of the report the reviewer says: "Although the majority report claims that on the substantial point a dissenting minority report by Professor Bolton is in accord with their own, we do not think our readers will agree with them." Prof. Bolton's report was not a "dissenting" report. On the contrary as it plainly shows, it was written at least two months in advance of the majority report. Prof. Bolton reported individually, not because he differed from his colleagues, but because he was obliged to go to Europe and could not remain until his colleagues' report was completed. He specifically states that he believes "that the conclusions I (he) have been able to reach do not differ essentially from those of the other members of the commission."

Now, let us see whether the reviewer is correct when he says that the board's report "does not venture to touch on most of the disputed points." Take the question of credit due Dr. Detmers for the discovery of the hog-cholera germ, of which the reviewer and Dr. Billings both make so much. The majority report says: "It is the opinion of the Commission that the microbe that Dr. Detmers at present regards as the specific cause of 'hog-cholera' is probably the same microbe which is considered by the Bureau authorities as the specific cause of hog-cholera; but according to present requirements of bacterial research and interpretation, it is impossible to declare that the organism as described by him in his reports published by the Department of Agriculture was the same thing." Also, "it is the opinion of the Commission that the actual and undeniable proof of the pathogenic relations between the so-called 'hog-cholera' germ and the disease of hog-cholera was first published in the annual report of the Department of Agriculture for 1855, and in the second annual report of the Bureau of Animal Industry for the same year, hence was not antedated with respect to epidemic diseases of swine existing in the United States."

Compare the minority report which reads, "I believe that Dr. Detmers would readily acknowledge that we can only be sure in such cases when we can isolate the organism and reproduce the disease with the organism so isolated, and it is not to his discredit that he was unable with the methods then employed to do this." Also, "I have not been able to find that the descriptions of the germs contained in the above mentioned reports from the Bureau have been antedated by other correct descriptions." Does the reviewer need any plainer language than that?

Then as regards the methods of scientific work in the Bureau of Animal Industry, which were called in question by Billings and by the reviewer. The majority report says: "In their observations of the methods of bacteriological research pursued by the Bureau of Animal Industry at Washington, the Commission are of the opinion that as to carefulness and precision they are up to the standard of modern requirements concerning bacteriological investigations. The minority report says: "It is only by the correct application of Koch's method that trustworthy results can be obtained, and it does not appear that these methods are employed in any investigations previous to the Bureau reports."

Now, take the question of there being two diseases, and the majority report says: "It is the opinion of the Commission, based upon their own individual observations and examinations of the subject, that there are at least two wide-spread epidemic diseases of hogs in this country, which are caused by different micro-organisms." The minority report says: "During my work as commissioner I have failed to meet with an epizootic which I am satisfied was what is termed 'swine-plague' in the Bureau reports, though previous to my appointment on the Board I studied one such outbreak . . . . . . In my investigations as commissioner I have been able to find but one organism which, in my opinion, caused the outbreaks under examination, and that I regard as identical with the hog-cholera germ described in the report of the Bureau, and I find the description therein given correct."

Those are the chief disputed points, and they are covered as definitely as any unbiased person could ask. They are exactly the points which Billings clamored to havs decided, and if the commission decided them coriectly the case of both Billings and the reviewer goes to pieces. The very germ which Billings declared was a forgery, and which he asserted did not exist in any form of American swine-plague, viz., the hog-cholera germ, was found in his possession and was given by him to the Commission as the germ of the disease he had been studying. That germ is so different from the *Schweineseuche* germ that the merest tyro in bacteriological study should be able to distinguish them; and yet for two yeais Billings had been claiming their morphological identityand applying the description of the *Schweineseuche* germ to the bacillus of hog-cholera.

Fortunately we now have the additional testimony of Prof.

Welch on the disputed points. Although declining to serve on the board he continued his investigations of swine-diseases, and has lately made the following statement of his results :—

THE JOHNS HOPKINS HOSPITAL, North Broadway,
BALTIMORE, November 8th, 1889.

DR. D. E. SALMON, Chief Bureau of Animal Industry,
WASHINGTON, D. C.

*Dear Sir* :—I am happy to comply with your request to report to you some results of our investigations of the epidemic diseases of swine known as hog-cholera, swine-plague, pneumo-enteritis, etc.

In the bacteriological examination of a large number of cases in various epidemics of this disease we have found two micro-organisms with such frequency as to lead us to believe that they bear a causative relation to the affection. These organisms in accordance with the nomerclature which you have adopted are the hog-cholera bacillus and the swine plague bacillus.

The hog-cholera bacillus we have found in a large proportion, although not in all cases. It has been present in the blood, lungs, spleen, kidneys, intestine and other parts. It is a bacillus, on the average somewhat longer than the swine-plague bacillus, which grows readily on all of the ordinary culture media. On potato its growth presents usually a brownish tint. It is actively motile. In the tissues it can be made in many instances to assume the peculiar stain which you have described, namely a nearly uniformly stained margin with a pale center. It is pathogenic for mice, rabbits, guinea pigs, and in sufficient doses for pigeons. The duration of life after inoculation of mice and rabbits is usually from five to fourteen days, and it may be even longer. The most important lesions in these inoculated animals are enlarged spleen, peculiar necrotic foci in the liver and fatty degeneration of the heart. Pure cultures of this bacillus when fed to pigs or when injected into the duodenum of pigs cause death with diphtheritic inflammation and necrosis of the large intestine.

It is apparent from the foregoing description that we have found the properties of the hog-cholera bacillus, such as were described in the Annual Report of the Bureau of Animal Industry for the year 1885. The description there given we regard as correct in its essential features and sufficient for the identification of the organism by other investigators. So far as we can learn, no description of this organism has been given prior to the appearance of the above mentioned report, certainly none sufficing for its identification.

The second organism which we have found in our investigations, is the swine-plague bacillus. This we have met with also in a large number of cases. It is found most frequent in the lungs when the seat of pnuemonia, but it may be present in the spleen, liver, intestine and other parts. We have not been able to determine any uniform anatomical differences between the cases in which the swine-plague bacillus aolne has been present and those cases in which only the hog-cholera bacillus has been found. Not infrequently both the swine-plague and the hog cholera bacillus have been present in the same cases.

The swine-plague bacillus in a short, oval bacterium, averaging 0.6 to 0.8 m. in length. It grows on most of our ordinary culture media, but an potato we have not been able to make it grow for more than one or two generations, and then the growth is invisible and probably due to the transference of a little nutritive medium from the original culture to the potato. It is not motile. It is a polar staining organism. It is pathogenic for mice, rabbits, guinea pigs and pigeons. We have found at least two varieties of this organism as regards virulence, the one killing rabbits in from 16 to 24 hours with abundant multiplication of the organism in the blood, and the other killing rabbits in from two to six days with extensive local lesions at the seat of inoculation, and with peritonitis, but often with few or no bacilli in the blood. The swine-plague bacillus is pathogenic for swine. When injected into the lungs it is capable of producing pneumonia and pleurisy, and often pericarditis. It is also capable of causing peritonitis.

It is apparent that the foregoing description is in accordance with the results which you have obtained with this organism, and which were published in the Report of the Bureau of Animal Industry for 1886, and in that of the Department of Agriculture for 1887. This organism corresponds in its properties to that described previously by Löffler and by Schütz as the bacterium of *Schweineseuche.*

Dr. F. S. Billings has kindly sent me a number of cultures of the micro-organism which he has obtained from swine in Nebraska, and which he regards as the cause of the disease called by him swine-plague. These cultures, which were for the most part pure, were of the bacillus described by you as the bacillus of hog-cholera and so designated in this letter. I failed to find the swine-plague bacillus (as described by you and in this letter) in Dr. Billings' cultures. We have had an opportunity of examining cultures of the *Schweineseuche* bacterium obtained from Koch's laboratory in Berlin, and we find that this is not identical with the bacillus found in Dr. Billings cultures."

Yours very Respectfully,

WILLIAM H. WELCH.

It will be seen by the above letter that the conclusions of the eminent pathologist of Johns Hopkins University are completely in accord with those of the Bureau of Animal Industry. He has found two diseases. The germs to correspond with our descriptions. He regards our descriptions as entitled to priority. He decides that Billings was working with the hog-cholera germ, and after comparing it with the *Schweineseuche* germ, he declares positively that they are not identical.

The evidence which I have referred to in this communication ought to be sufficient to settle any question of this kind, and it will settle it with those who are interested in the matter as a question of science, but those who are actuated by motives of revenge and jealously will probably continue to dispute, and will throw mud at every scientist, no matter how eminent he may be, who differs from their expressed opinions.

Before closing, there is just one other misstatement made by the reviewer which I will correct, and then I will leave the question to the judgment of your impartial readers. The reviewer says: "Some inoculated hogs were placed at the disposal of the committee by Dr. Billings. The former report that these hogs withstood every test they were put to, but they do not say what 'tests'." The Board nowhere say that the hogs withstood every test. They did not go into the details of their experiments in their report. On the contrary I have in my possession a written statement from the chairman of the Board, who made these experiments, to the effect that these hogs when exposed became very sick; and although none of them died, it is equally true that none of the uninoculated animals used as checks died. If Billing's inoculated hogs became very sick from an exposure which did not kill unprotected animals it would be absurd to say that they withstood every test.

I will not take more of your space to point out the other inaccuracies and distortions of facts which are found in the review under consideration. Going over those already mentioned the reader will undoubtedly be astonished that any one with a reputation at stake could be found who either through carelessness or malice would make such misrepresentations, and he will be led to ask if the character of the paper is an explanation of the absence of the author's signature?

D. E. SALMON.

